# DNA Methylation Associates With Sex‐Specific Effects of Experimentally Increased Yolk Testosterone in Wild Nestlings

**DOI:** 10.1111/mec.17647

**Published:** 2025-01-06

**Authors:** Bernice Sepers, Suvi Ruuskanen, Tjomme van Mastrigt, A. Christa Mateman, Kees van Oers

**Affiliations:** ^1^ Department of Animal Ecology Netherlands Institute of Ecology (NIOO‐KNAW) Wageningen The Netherlands; ^2^ Behavioural Ecology Group Wageningen University & Research (WUR) Wageningen The Netherlands; ^3^ Department of Evolutionary Population Genetics Bielefeld University Bielefeld Germany; ^4^ Department of Biological and Environmental Science University of Jyväskylä Jyväskylä Finland; ^5^ Department of Biology University of Turku Turku Finland; ^6^ Vogeltrekstation – Dutch Centre for Avian Migration and Demography Netherlands Institute of Ecology (NIOO‐KNAW) Wageningen The Netherlands

**Keywords:** androgens, behaviour, development, epigenetics, maternal effects, sex‐specific effects

## Abstract

Maternal hormones can profoundly impact offspring physiology and behaviour in sex‐dependent ways. Yet little is known about the molecular mechanisms linking these maternal effects to offspring phenotypes. DNA methylation, an epigenetic mechanism, is suggested to facilitate maternal androgens' effects. To assess whether phenotypic changes induced by maternal androgens associate with DNA methylation changes, we experimentally manipulated yolk testosterone levels in wild great tit eggs ( *Parus major* ) and quantified phenotypic and DNA methylation changes in the hatched offspring. While we found no effect on the handing stress response, increased yolk testosterone levels decreased the begging probability, emphasised sex differences in fledging mass, and affected methylation at 763 CpG sites, but always in a sex‐specific way. These sites are associated with genes involved in growth, oxidative stress, and reproduction, suggesting sex‐specific trade‐offs to balance the costs and benefits of exposure to high yolk testosterone levels. Future studies should assess if these effects extend beyond the nestling stage and impact fitness.

## 1 Introduction

Environmental conditions experienced by mothers can have profound impacts on the phenotypes of their offspring (Mousseau and Dingle 1991; Bernardo 1996; Kofman 2002; Groothuis et al. 2005; Maestripieri and Mateo 2009). These so‐called environmental maternal effects are aspects of the maternal phenotype that cause changes in offspring phenotype and have been documented across a wide array of taxa (e.g., Räsänen and Kruuk 2007). Maternal effects are predicted to adaptively modulate offspring phenotypes according to the local environment (Mousseau and Fox 1998; Marshall and Uller 2007; Yin et al. 2019; Sánchez‐Tójar et al. 2020). Females may influence their offspring prenatally through, for example, incubation temperature (Hepp, Kennamer, and Johnson 2006; Mitchell, Maciel, and Janzen 2015) and by providing nutrients (Roseboom, de Rooij, and Painter 2006; de Rooij et al. 2010) or other resources, such as antioxidants (Blount et al. 2002), immune factors (Blount et al. 2002; Saino et al. 2002), and hormones (Schwabl 1993; McCormick 1999; Dloniak, French, and Holekamp 2006; Uller, Astheimer, and Olsson 2007; Dantzer et al. 2013). These prenatal conditions can subsequently affect offspring sex ratios (e.g., Carter, Bowden, and Paitz 2017), development (Hepp, Kennamer, and Johnson 2006; Mitchell, Maciel, and Janzen 2015), physiology (Blount et al. 2002; Saino et al. 2002; Boots and Roberts 2012), behaviour (Eising, Muller, and Groothuis 2006; Partecke and Schwabl 2008), and ultimately offspring fitness (e.g., Ruuskanen et al. 2012). What mediates the effects of prenatal conditions has, however, remained elusive (e.g., Groothuis et al. 2019; Bebbington and Groothuis 2021).

Maternal hormones can profoundly affect offspring phenotypes. In oviparous species, the effects of yolk androgen hormones, especially testosterone, on phenotypic traits of developing offspring have been extensively studied (Gil 2008). Yolk testosterone levels have been found to vary between clutches, which can partly be explained by between‐female differences, such as female age (Pilz et al. 2003), perceived predator risk (Coslovsky et al. 2012), and personality (Ruuskanen et al. 2018), and by environmental conditions, such as parasite load (Tschirren, Richner, and Schwabl 2004), or social conditions, such as mate attractiveness (Remeš 2011) and breeding density (Groothuis and Schwabl 2002; Eising et al. 2008; Remeš 2011; Bentz, Navara, and Siefferman 2013). Despite studies showing that testosterone and other yolk androgens have largely disappeared after five days of incubation (Kumar et al. 2019), yolk testosterone has profound effects on offspring physiology and behaviour. Increased yolk testosterone levels have been found to stimulate nestling growth (Schwabl 1996; Navara, Hill, and Mendonça 2005, 2006; Müller et al. 2007), decrease social dependence and neophobia (Daisley et al. 2005), and alter breath rate (Bentz, Navara, and Siefferman 2013), food intake, and begging rates (Schwabl 1996; Eising and Groothuis 2003).

The effects of increased yolk testosterone levels are not always in the same direction, as some studies have reported decreased or unaffected begging rates (Pilz et al. 2004; Boncoraglio et al. 2006) and reduced growth (Sockman and Schwabl 2000; Rubolini et al. 2006). Moreover, yolk testosterone can be deposited in a sex‐specific way (Müller et al. 2002) and is known to induce sex‐specific effects (Tschirren 2015). Yolk testosterone has, for example, been found to promote growth in male offspring, while female growth was unaffected (Tschirren 2015) or reduced (Saino et al. 2006). Likely, developmental trajectories and their associated costs and fitness returns are differentially affected in male and female offspring (Gil 2008). This suggests a pivotal role for yolk testosterone in explaining how the sexes trade off developmental processes differently. However, the interpretation of such sex‐specific effects might be improved if the underlying molecular mechanisms that allow for such sex‐specific effects are taken into account (Gil 2008; Groothuis Ton and Schwabl 2008). Yet little is known about the molecular mechanisms that are responsible for these phenotypic changes in developing offspring caused by variation in yolk testosterone.

Epigenetic mechanisms have been suggested to facilitate effects of maternal androgens (Groothuis Ton and Schwabl 2008), specifically DNA methylation (Sepers et al. 2019). DNA methylation, the addition of a methyl group to a DNA nucleotide, interferes with the binding of transcription factors to the DNA (Bird 2002; Moore, Le, and Fan 2013; Yin et al. 2017). DNA methylation usually suppresses gene expression (Bird 2002; Goldberg, Allis, and Bernstein 2007; Moore, Le, and Fan 2013), specifically if methylated cytosines in CpG context (CG dinucleotides) are located nearby the transcription start site of a gene (Bird 2002; Goldberg, Allis, and Bernstein 2007; Li et al. 2011; Moore, Le, and Fan 2013; Laine et al. 2016). Therefore, DNA methylation is generally expected to affect the expression of phenotypic traits (Law and Jacobsen 2010). Prenatal maternal effects on DNA methylation have been found in the offspring of vertebrates such as mice (St‐Cyr and McGowan 2015) and humans (Tobi et al. 2009, 2014). Studies in wild avian species have experimentally shown (Hukkanen et al. 2023) or suggested (Bentz et al. 2016) that maternal androgens can impact offspring methylation. To our knowledge, there is only one genome‐wide study on the effects of experimentally elevated yolk androgens (in this case testosterone) on DNA methylation. In this study on zebra finches ( *Taeniopygia guttata* ), differentially methylated regions between treated and untreated individuals were found in or near genes that were also differentially expressed in the hypothalamus and amygdala (Bentz et al. 2021). However, this might be specific to males, as female zebra finches were not included in this study, and sex differences in DNA methylation are known to exist in several bird species. Multiple cytosines on the Z chromosome and on chromosome 1 are differentially methylated between the sexes in chickens (Natt, Agnvall, and Jensen 2014) and in several other species belonging to the order Galliformes (Teranishi et al. 2001), suggesting that differences in DNA methylation might aid in phenotypic sex differentiation. For example, CpG sites in a 460‐kb long region on the short arm of the Z chromosome are hypermethylated in male chicken embryos compared to female embryos (Teranishi et al. 2001). Furthermore, CpG sites in the *dopamine receptor D4* gene are significantly hypermethylated in female great tits compared to males (Verhulst et al. 2016), but not in other genes (Sepers, Chen, et al. 2023), suggesting that sex‐specific DNA methylation patterns can be gene‐ or region‐specific. While these studies clearly indicate that DNA methylation patterns cannot be generalised across sexes, it remains largely unknown to what extent DNA methylation is an underlying mechanism for yolk testosterone‐mediated sex‐specific maternal effects.

Here, we experimentally manipulated yolk testosterone levels in a population of wild great tits ( *P. major* ) and assessed pre‐fledging effects on biometric measures, behavioural traits, and genome‐wide DNA methylation to test whether yolk testosterone‐induced changes in DNA methylation may explain sex‐specific phenotypic effects. We expected that testosterone positively affects body mass and tarsus length in male great tits, but not in females (Tschirren 2015), explained by higher competitiveness in testosterone‐treated males, observed by a higher begging rate and higher food reception (Schwabl 1996; Eising and Groothuis 2003). We expected that, as a result of experiencing favourable nutritional conditions (van Oers et al. 2015), testosterone‐treated males would be more resilient to stress and therefore show a lower handling stress response than testosterone‐treated females. As a low handling stress response is associated with decreased levels of exploratory behaviour after fledging (Fucikova et al. 2009), this lower reactivity to acute stress might reflect behavioural adaptation to a non‐competitive environment. If sex‐specific effects of testosterone on behaviour and biometry are mediated by DNA methylation, we expected the transcription start site regions of genes involved in sexual dimorphism, hormone receptor expression, steroid hormone secretion, neurone development, morphology, and growth to be differentially methylated between the testosterone‐treated group and the control group, but only in within‐sex comparisons. Our results show that sex‐specific DNA methylation changes associate with sex‐specific effects on biometry and behaviour caused by experimentally induced elevated yolk testosterone levels. This therefore strongly suggests that DNA methylation is indeed a mediator of post‐hatching sex‐specific effects of elevated yolk testosterone on offspring phenotypes, but these effects do not always induce a more competitive phenotype pre‐fledging.

## 2 Materials and Methods

This study was conducted in 2020 in a long‐term nest box population in the Westerheide estate, near Arnhem, the Netherlands (52°01′00″ N, 05°50′30″ E). All information regarding the number of eggs, nestlings, clutches, and broods is provided in Tables S1, S2 and S5.

### 2.1 Experimental Injection Protocol

We manipulated yolk androgen levels following a procedure that has been successfully applied before (e.g., Tschirren et al. 2005; Ruuskanen and Laaksonen 2010) (described in detail in Appendix S1A). Briefly, clutches were alternately assigned to be in either the testosterone‐treated group or the control group. On the day the fifth egg was laid, egg yolks in clutches assigned to the testosterone‐treated group were injected with 12 ng of testosterone dissolved in 5 μL of sesame oil, while egg yolks in control clutches were injected with 5 μL of sesame oil. We aimed to elevate yolk testosterone to concentrations that would still fall within the population's natural range in the majority of eggs while inducing detectable effects on biometry (Tschirren 2015). We therefore injected 12 ng testosterone per egg (approximately twice the standard deviation of yolk testosterone concentrations in great tit eggs from a nearby study population: mean ± SD = 22.9 ± 6.2 ng/yolk in Ruuskanen, Darras, de Vries, et al. 2016). Thereafter, injections were done each day for the newly laid egg. Clutch size, egg weight, the duration of the incubation period, and hatching success did not differ significantly between the treatment groups (Appendix S2A). Although hatching success was low (58.4% for control broods and 61.6% for testosterone‐treated broods, Table S1), it was high compared to other injection studies (50%–55%, e.g., Podlas, Helfenstein, and Richner 2013; Ruuskanen, Darras, Visser, et al. 2016; Bentz et al. 2021).

### 2.2 Cross‐Fostering

To minimise the differences in rearing environment between nestlings from testosterone‐treated eggs and nestlings from control eggs, we used a partial cross‐foster design as in Van Oers et al. (2015) and Sepers et al. (2021) (described in detail in Appendix S1B). Briefly, we assigned broods to pairs on day 2 or 3 after hatching. Within a pair, the nestlings were weighed (to the nearest 0.01 g) and partially cross‐fostered to create mixed broods containing nestlings from both treatments. Pairs of small broods (± three nestlings) were merged into one brood to lower the chance of desertion. We discarded 21 cross‐fostered broods where the testosterone‐treated nestlings were one day younger than the control nestlings from all analyses (asynchronous pairs, Table S1), as this made it impossible to disentangle treatment effects from age effects. Therefore, all analyses were only conducted on cross‐fostered broods with zero days difference in hatching date between the control and the testosterone‐treated nestlings (synchronous pairs, Table S1). On day 6 after hatching, the nestlings were weighed, and approximately 10 μL of blood was collected by brachial venipuncture. The samples were stored at room temperature in 1 mL of cell lysis buffer (Gentra Puregene Kit, Qiagen, USA) until further analysis for molecular sexing (following Griffiths et al. 1998) and to measure erythrocyte DNA methylation levels.

### 2.3 Video Recordings and Analysis

Seven days after hatching, we installed an infrared spy camera (Velleman, CMOS camera, CAMCOLMBLAH2) to record nestling food solicitation behaviours. Cameras were connected to a digital video recorder placed outside the nest box (PV‐500L2, LawMate International, Taipei, Taiwan). On day 8 after hatching, we weighed nestlings and marked them with red acrylic paint to enable their identification on video recordings under infrared light. Subsequently, we recorded the brood for at least 2 h between 07:00 and 15:00. One nest box was recorded between 15:30 and 17:30. At least 1.5 h of video recordings were analysed using Adobe Premiere Pro 2021 (Adobe Inc.) by a single person who was blind with respect to nestling treatments. During each parental feeding visit, we scored (1) which nestlings showed a begging response right before feeding and (2) which nestling received food.

### 2.4 Handling Stress Test

We conducted a handling stress test 14 days after hatching, as described in Fucikova et al. (2009) and Sepers, Mateman, et al. (2023), with two modifications. We measured handling stress for only 1 min instead of 2, and the nestlings were not socially isolated. Handling stress was measured by counting the number of breath movements (i.e., breath rate) during four subsequent bouts of 15 s each. Once all nestlings were tested separately, they were weighed, and their tarsus length (callipers, ± 0.1 mm) was measured. Individual estimates for the handling stress response were obtained as described in Sepers, Mateman, et al. (2023), using a linear mixed model (LMM) with the number of breaths per 15 s bout as the dependent variable. The produced estimates (i.e., handling stress response) quantify the individual deviations from the average slope in breath rates over time and were extracted for further analysis.

### 2.5 DNA Methylation Data Generation and Bioinformatics

From all blood samples collected on day 6 after hatching, we randomly selected four samples per brood of rearing (two samples from each treatment). In total, we selected 180 samples, of which 100 were from broods without an age difference between control and testosterone‐treated nestlings (Table S5). We assessed genome‐wide DNA methylation levels using epiGBS2 (Gawehns et al. 2022). This is a reduced‐representation DNA methylation laboratory protocol and a bioinformatics pipeline. Five libraries, each containing 36 barcoded samples, were prepared and sequenced as described in Gawehns et al. (2022) with the improvements reported in Sepers, Mateman, et al. (2023) and described in detail in Appendix S3.

Raw reads were demultiplexed, checked, trimmed, filtered, merged, aligned, and called for methylation using the reference branch of the epiGBS2 bioinformatics pipeline (Gawehns et al. 2022) with minor modifications. We aligned the reads in directional and paired‐end mode to the *P. major* reference genome v1.1 (GCF_001522545.3) (Laine et al. 2016), removed overlap between read pairs (Table S6), and called methylation in CpG context only. We observed a bias in DNA methylation due to read position, which could reflect sequencing errors, base‐calling errors (Hansen et al. 2012; Taub et al. 2010), or introduced cytosines during end‐repair (Bock 2012). Therefore, we ignored the first four nucleotides in all reads. Mapping efficiency ranged from 43.10% to 51.40% (Table S6). While mapping efficiency seems low, it is comparable to other bisulfite sequencing studies in great tits (e.g., Mäkinen et al. 2019; van Oers et al. 2020; Sepers, Chen, et al. 2023; Sepers, Verhoeven, and van Oers 2024). Complementary CpG dinucleotides were merged using the R package *methylKit* v1.16.1 (Akalin et al. 2012). Subsequently, using a custom script, CpG sites (CpGs) with low coverage (< 10×) or very high coverage (> 99.9th percentile) and CpGs that were not covered in at least 15 individuals in each treatment or with a high (< 0.05) or low (> 0.95) mean methylation level across all individuals were discarded (Table S7), leading to a total of 169,215 CpGs in the final analysis.

### 2.6 Statistical Analysis

#### 2.6.1 General

For all mixed models described below, unless described otherwise, we included treatment, sex, and their interaction as fixed effects. Brood of origin and brood of rearing were included as random effects to account for the non‐independence of nestlings from the same brood.

We used a binomial error distribution and logit‐link function for all generalised linear mixed models (GMMLs). The significance of the interaction of treatment with sex and/or the significance of treatment was determined with a Likelihood Ratio Test (LRT) by comparing a model with the interaction or factor of interest with a model without the interaction or factor of interest using the *anova* function in R.

All LMMs were run with ML estimation, and we used backwards elimination of the interaction between treatment and sex based on the p‐values provided by a type III analysis of variance via Satterthwaite's degrees of freedom method using the *anova* function in R. In case the interaction was non‐significant (*p* < 0.05 for biometry and behavioural data, *p* < 0.1 for DNA methylation data), it was deleted. The minimal adequate model always included treatment and sex. The analyses were done using the packages *lme4* v1.1.28 and *lmerTest* v3.1.3. Post hoc comparisons were performed with the *lsmeans* function in the package *emmeans* v1.7.2. *p*‐values were provided via the Satterthwaite's degrees of freedom method and corrected for multiple testing with a Bonferroni correction.

All behaviour and biometry analyses were done in RStudio v2021.9.2, while the analyses of the methylation calls were done with RStudio v1.4.1717.

#### 2.6.2 Statistical Analysis, Biometry and Behaviour

We used separate LMMs to analyse the effect of the treatment on weight on day two, day 6, day 8, and day 14 after hatching and on tarsus length and handling stress on day 14. Brood of rearing was not included when analysing the effect on weight on day 2, as weighing happened before cross‐fostering.

We analysed the effect of the treatment on the probability of begging (yes/no) and the probability of getting fed at each parental visit using GLMMs. Nestling ID was included as a random effect in addition to the fixed and random effects described in *general*.

#### 2.6.3 Statistical Analysis of DNA Methylation

To analyse the effect of the treatment on DNA methylation level per CpG, we used a GLMM in which the dependent variable was modelled as the fraction of the number of methylated Cs over the total number of analysed reads (i.e., coverage: number of methylated Cs plus unmethylated Cs per CpG) with the *cbind* function. This model was run for each CpG separately and included the fixed and random effects described in *general*. CpGs with a False Discovery Rate (FDR) (Benjamini and Hochberg 1995) corrected *p*‐value below 0.1 were considered as significantly differentially methylated CpGs. CpGs for which the effect of the treatment depended on sex were referred to as sex‐specific differentially methylated sites (sex‐specific DMS). We excluded models of CpGs that produced warnings other than singularity warnings. Furthermore, we corrected for potential overdispersion by excluding CpGs that fell out of the 95% Highest Density Interval (HDI) for the distribution of the dispersion statistic (Zuur, Hilbe, and Ieno 2013) using the R package *HDInterval* v0.2.2 (Table S7).

Subsequently, each sex‐specific DMS was assigned to a category. A significant (FDR corrected) difference between control and testosterone‐treated females only corresponded to the category “female‐specific DMS”, while a significant difference between control and testosterone‐treated males only corresponded to the category “male‐specific DMS”. A significant difference between control and testosterone‐treated individuals in both the females and males, while the within‐sex differences were in opposite directions, corresponded to the category “antagonistic DMS”. No significant difference between the treatments in either the males or females, but some other significant difference corresponded to the category “other DMS” (e.g., significant difference between testosterone‐treated females and control males).

#### 2.6.4 Gene Annotation and Ontology Analyses

CpGs were assigned to genomic regions using custom R scripts, R packages *GenomicFeatures* v1.42.3 (Lawrence et al. 2013) and *rtracklayer* v1.50.0 (Lawrence, Gentleman, and Carey 2009), and the *P. major* reference genome build v1.1, annotation version 102 (Laine et al. 2016). CpGs were assigned to the following genomic regions: TSS region (300 bp upstream to 50 bp downstream of the annotated transcription starting position), promoter region (2000 bp upstream to 200 bp downstream of the annotated transcription starting position), gene body (intron or exon), upstream (10 K bp upstream of the gene body), and downstream (10 K bp downstream of the gene body) (Laine et al. 2016; Viitaniemi et al. 2019; Lindner, Laine, et al. 2021; Lindner, Verhagen, et al. 2021; Sepers et al. 2021). If a CpG was assigned to multiple regions, the regions were prioritised in the above‐described order. If a CpG was assigned to both up‐ and downstream regions, we prioritised characterised genes and genes with the nearest gene body. To facilitate interpretation of genes associated with these sex‐specific DMS, we searched the literature for relevant studies on phenotypic effects, DNA methylation, and gene expression. We specifically focused on genes with DMS within regulatory regions (promoter and TSS regions) and (groups of) genes with multiple DMS (Tables S17–S20). In addition, we identified enriched Gene Ontology (GO) terms using the ClueGO v2.5.8 (Bindea et al. 2009) plug‐in for Cytoscape v3.9.1 (Shannon et al. 2003) following Sepers, Verhoeven, and van Oers (2024). The target lists consisted of all genes associated with either (1) antagonistic DMS, (2) female‐specific DMS, or (3) male‐specific DMS. The background list consisted of all genes associated with any of the analysed CpGs, except for 3603 functionally uncharacterised genes (i.e., LOC genes). All ontologies were updated on 09‐03‐2022. We used Revigo (Supek et al. 2011) to summarise GO terms and eliminate redundant terms. We applied a semantic similarity cut‐off value of 0.5 *sim*_*Rel*_ (Schlicker et al. 2006).

## 3 Results

### 3.1 Treatment Effects on Biometry

Nestlings hatching from testosterone‐injected eggs did not differ in weight from those hatching from control eggs on day 2 (treatment, LMM: *F*_1,34.10_ = 0.30, *p* = 0.59; Table S8), day 6 (treatment, LMM: *F*_1,33.19_ = 0.13, *p* = 0.72; Table S9), or day 8 after hatching (treatment, LMM: *F*_1,27.43_ = 0.26, *p* = 0.61; Table S10). Sexes significantly differed in weight on day 6 after hatching (sex, LMM: *F*_1,137.06_ = 5.43, *p* = 0.02), but not on day 2 or 8 (all *p* ≥ 0.25). The treatment effect on variation in weight on day 2, 6, or 8 did not differ between the sexes (treatment × sex, all *p* ≥ 0.22).

**FIGURE 1:**
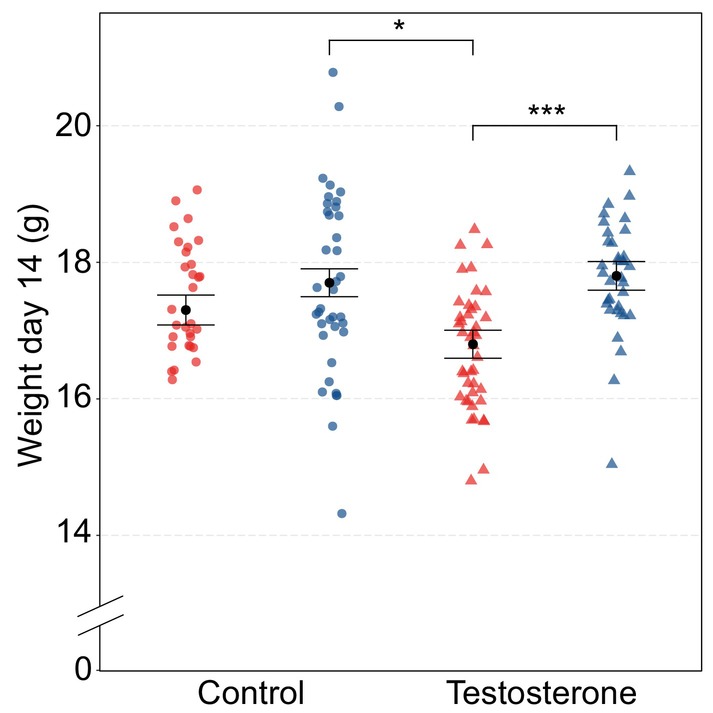
Weight day 14. Weight (g) on day 14 after hatching for both sexes and control and testosterone‐treated treated nestlings. Red dots and triangles represent raw data points for females. Blue dots and triangles represent raw data points for males. Black dots represent the predicted marginal means of both sexes in both treatments. Error bars represent standard error of predicted marginal means. *P*‐values below 0.05 but above 0.01 are indicated with *, *p*‐values below 0.001 are indicated with ***.

Testosterone treatment affected nestling weights on day 14 after hatching in a sex‐specific way (treatment × sex, LMM: *F*_1,110.24_ = 5.24, *p* = 0.02; Table S11). Testosterone‐treated females weighed significantly less than testosterone‐treated males (estimate ± SE = −0.98 ± 0.17, *t*‐ratio_114.5_ = −5.64, *p* < 0.001; Figure 1), while the sexes in the control group did not differ significantly in weight (−0.41 ± 0.18, *t*‐ratio_114.2_ = −2.28, *p* = 0.15), and treatment effects within sexes were non‐significant (females: 0.48 ± 0.27, *t*‐ratio_32.2_ = 1.74, *p* = 0.55; males: −0.09 ± 0.27, *t*‐ratio_30.3_ = −0.33, *p* = 1.00). We found a non‐significant trend (treatment, LMM: *F*_1,27.54_ = 3.08, *p* = 0.09; Table S12) that testosterone‐treated individuals (mean ± SE = 19.30 ± 0.07) had longer tarsi on day 14 after hatching compared to control individuals (19.10 ± 0.07), after correcting for sex differences (sex, LMM: *F*_1,127.72_ = 61.30, *p* < 0.001). This tendency existed irrespective of the offspring sex (treatment × sex, LMM: *F*_1,120.93_ = 0.40, *p* = 0.53).

### 3.2 Treatment Effects on Behaviour

**FIGURE 2:**
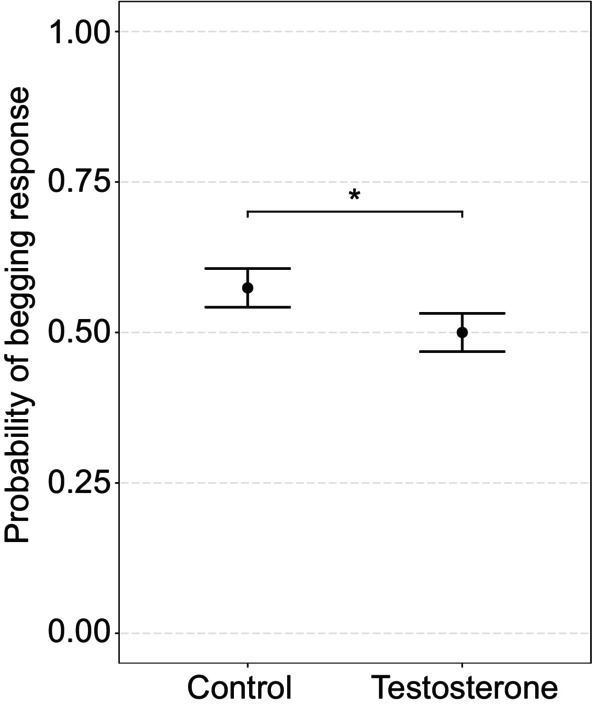
Begging probability. The effect of treatment on the average probability (± SE) of begging during parental visits. *P*‐value below 0.05 but above 0.01 is indicated with *.

Testosterone‐treated nestlings (probability ± SE: 0.50 ± 0.03) begged significantly less than control nestlings did (0.57 ± 0.03) (treatment, GLMM: $\chi_{1}^{2}$ = 3.99, *p* < 0.05; Figure 2; Table S13). This did not result in different probabilities of being fed (treatment, GLMM: $\chi_{1}^{2}$ = 1.02, *p* = 0.31; Table S14). The treatment effect on the probability of begging or getting fed did not differ between the sexes (treatment × sex, all *p* ≥ 0.18; Figure S1). Sex differences in the handling stress response (sex, LMM: *F*_1,124.63_ = 10.38, *p* = 0.002; Table S15) were not affected by the treatment (treatment × sex, LMM: *F*_1,122.21_ = 1.90, *p* = 0.17). Treatment itself also did not affect the handling stress response (treatment, LMM: *F*_1,32.85_ = 0.006, *p* = 0.94) when correcting for sex.

### 3.3 Treatment Effects on DNA Methylation

**FIGURE 3:**
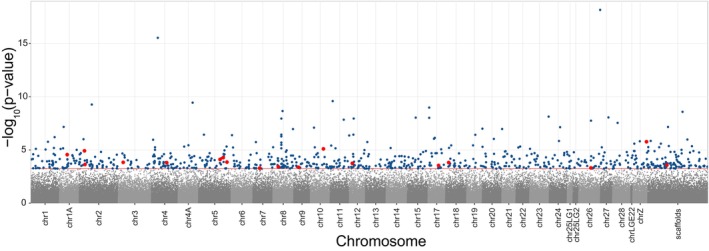
Manhattan plot of sex‐specific DMS. Manhattan plot showing the significance of the interaction of treatment (control and testosterone) with sex in explaining variation in DNA methylation (−log_10_(*p*‐values)). Each dot represents a CpG tested for a sex‐specific treatment effect (169,215 CpGs). Dark blue dots represent CpGs with a significant sex‐specific treatment effect. Red dots represent significant CpGs that are in the TSS region of a gene. The dotted red line marks the genome‐wide significance threshold. The sites are plotted against the location of the associated site within the genome. Alternating colours help to differentiate adjacently displayed chromosomes. ChrZ is a sex chromosome; all the other chromosomes are autosomes. All unplaced scaffolds are merged into the category scaffolds.

While we did not find any DMS when comparing methylation between nestlings from testosterone‐treated eggs and control nestlings (all FDR‐corrected *p*‐values > 0.1), we found 763 sites that showed a sex‐specific treatment effect (Figure 3; Table 1). In 338 CpGs, testosterone significantly affected methylation in both sexes, but in different directions (antagonistic DMS). In 117 CpGs, we found a significant effect of testosterone on methylation in females, but not in males (female‐specific DMS), while the opposite pattern was found in 201 CpGs (male‐specific DMS).

**TABLE 1:** Number of sex‐specific DMS assigned to different categories.

	Female‐specific	Male‐specific	Antagonistic	Other	Total
Number of sex‐specific DMS	177	201	338	47	763

*Note:* The categories are based on whether the within‐sex differences between testosterone and control individuals are significant and the direction of the difference. Female‐specific DMS: FDR‐corrected *p*‐value < 0.1 between control and testosterone‐treated females only. Male‐specific DMS: FDR‐corrected *p*‐value < 0.1 between control and testosterone treated males only. Antagonistic DMS: A significant difference between the treatments in both sexes, while the within‐sex differences are in opposite directions. Other DMS: No significant difference between the treatments in either the males or females.

Out of these 763 CpGs, the 650 that could be annotated were found in or adjacent to 576 different genes, including 456 characterised genes. Out of the 650 annotated sex‐specific DMS, 138 were found in the promoter region of a gene, including 18 situated in the TSS region. Furthermore, 392 sites were found in a gene body, and 120 sites were found upstream or downstream of genes (Table S16).

**FIGURE 4:**
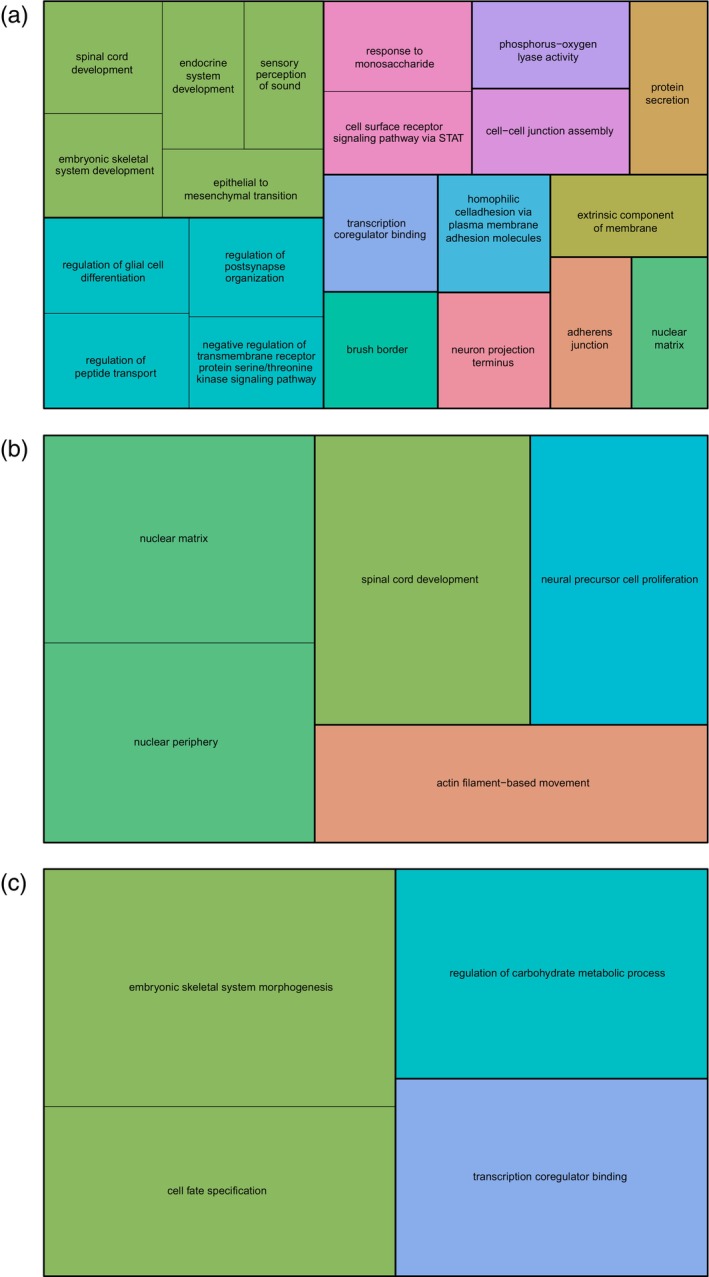
Representation of functional roles of genes associated with sex‐specific DMS. Treemaps of GO terms associated with sex‐specific DMS for the categories (a) antagonistic DMS, (b) female‐specific DMS and (c) male‐specific DMS. The GO terms from ClueGO were merged based on semantic similarity using REVIGO. Each rectangle indicates the FDR‐corrected *p*‐value of the representative GO term for each merged cluster. Within each category (i.e., treemap), different colours represent superclusters comprising related clusters within the ontologies of biological process, molecular function, and cellular component.

Using the genes associated with antagonistic DMS, we detected 49 significantly enriched GO terms (FDR corrected *p* < 0.05; Tables S21 and S22), which clustered into 21 enrichments. The gene ontologies were mainly involved in nervous system development, cell adhesion, protein secretion, regulation of signalling pathways, and transcription coregulator binding (Figure 4a).

We detected nine significantly enriched GO terms (FDR‐corrected *p* < 0.05) when we conducted a GO analysis on genes associated with female‐specific DMS only (Table S23). Five enrichments remained after merging. The gene ontologies were nuclear periphery, nuclear matrix, spinal cord development, neural precursor cell proliferation, and actin filament‐based movement (Figure 4b).

We also detected nine significantly enriched GO terms (FDR‐corrected *p* < 0.05) when we conducted a GO analysis on genes associated with male‐specific DMS only (Table S23), which merged into four enrichments. The gene ontologies were transcription coregulator binding, cell fate specification, embryonic skeletal system morphogenesis, and regulation of carbohydrate metabolic process (Figure 4c).

## 4 Discussion

Hormonally mediated maternal effects have profound effects on offspring phenotypes, often in a sex‐specific way. DNA methylation is predicted to mediate these effects. The aim of this study was, therefore, to assess whether DNA methylation can potentially mediate sex‐specific effects of experimentally manipulated yolk testosterone levels on postnatal biometry and behaviour. We found that experimentally elevated levels of yolk testosterone indeed affected individuals in a sex‐specific way. Increased levels of yolk testosterone decreased the probability of begging and increased fledging mass differences between the two sexes. Experimental exposure to high levels of yolk testosterone also affected DNA methylation at 763 CpGs. Interestingly, the direction of this effect always depended on offspring sex.

We found a similar number of male‐specific DMS and female‐specific DMS, suggesting that the sexes are equally sensitive to an increase in testosterone in terms of DNA methylation, but at different CpG sites. As DNA methylation can affect gene expression, it is generally expected to affect the expression of phenotypic traits (Law and Jacobsen 2010). Indeed, the biological functions of these genes were linked to phenotypic effects of experimentally elevated prenatal testosterone found in this study and in previous ones. However, we acknowledge that linking single differentially methylated sites to single phenotypic traits is challenging. First, the effect of DNA methylation outside of the promoter region on gene expression is not clear‐cut, and DNA methylation also affects the expression of distant genes, in so‐called trans effects (van Eijk et al. 2012). Second, gene expression variation itself is not easily translated into the expression of polygenic traits without knowing the underlying genetic architecture of the studied trait.

### 4.1 Nervous System Development and Behaviour

Female‐specific and antagonistic DMS were found in or near genes involved in nervous system development and behaviour (Table S17), which indicates that DNA methylation mediates sex‐specific effects of prenatal testosterone on brain organisation and behaviour. This was not reflected in the behavioural traits measured in our study, as we found no effect of exposure to high yolk testosterone on the handling stress response, and it decreased the probability of begging, but regardless of sex. As many of the female‐specific and antagonistic DMS were found in or near genes involved in cognitive phenotypes and neurological diseases (Table S17), it is possible that we should have focused on cognitive traits instead. Indeed, experimentally increased yolk testosterone affected the brain anatomy differently in male and female chickens (*Gallus domesticus*) (Schwarz and Rogers 1992), and in other bird species it has been found to affect lateral preference consistency (yellow‐legged gull, *Larus michahellis* : Possenti et al. 2016) and prenatal auditory learning (bobwhite quail, *Colinus virginianus* : Bertin et al. 2009), although regardless of sex. To our knowledge, there are no studies on the (sex‐specific) effects of experimentally elevated yolk testosterone on cognition in wild birds. Given the link of the differentially methylated genes in our study to cognition, we see ample opportunity for future experimental studies on the link between prenatal testosterone, epigenetic mechanisms, and avian cognition (Sepers et al. 2019).

Although most studies reported stimulating effects of experimentally increased yolk testosterone on begging behaviour (Schwabl 1996; Eising and Groothuis 2003), decreased begging rates as in our study have been found before as well (Pilz et al. 2004). As the treatment did not affect nestling mass on day 2, 6, or 8 after hatching, it is unlikely that a difference in nestling quality or the need for food explains the treatment effect on begging behaviour. It is more likely that this was facilitated by structural differences in nervous system development, possibly mediated by DNA methylation of genes involved in nervous system development and behaviour (Table S17).

### 4.2 Mass, Growth, and Metabolism

Exposure to high yolk testosterone increased fledging mass differences between the two sexes, since it had a negative effect on female but not on male fledging mass. However, this result must be interpreted with caution as the treatment only caused a marginally significant difference in fledging mass between females. This antagonistic effect of experimentally elevated yolk testosterone on growth has also been found in other bird species (Saino et al. 2006; Holmes and Schwabl 2022). Sex‐specific effects on fledging mass are also supported by the functions of several of the genes in which male‐specific and antagonistic DMS were found, such as growth and metabolism (Table S18), suggesting that the effect of prenatal testosterone on fledging mass is mediated by DNA methylation changes.

As described before, the decreased probability of begging of testosterone‐treated nestlings did not lead to a decreased nestling weight at earlier stages, possibly because sibling competition was low, which is supported by our results that they were not fed less. A likely explanation for the late appearance of an effect on weight is that when the nestlings from the testosterone group remain to beg less often than those from the control group when nestling competition increases, the female offspring may suffer the most in such suboptimal conditions (de Kogel 1997; Martins 2004). Indeed, several studies suggest that male great tit nestlings have a competitive advantage in suboptimal conditions (Dhondt 1970; Drent 1984; Smith, Kallander, and Nilsson 1989; Lessells, Mateman, and Visser 1996; Oddie 2000). However, we acknowledge that we cannot exclude the possibility that the effect on fledging weight was influenced by multiple or other factors, such as decreased energetic costs due to lower begging rates (which may be limited: Leech and Leonard 1997), higher digestive efficiency in testosterone‐treated males (Ruuskanen and Laaksonen 2013), or trade‐offs with, for example, unobserved physiological components (Moreno‐Rueda 2010).

### 4.3 Oxidative Stress

Since heavier fledglings are more likely to recruit locally (Tschirren 2015), the testosterone‐treated males in our study might be more likely to survive than testosterone‐treated females. However, elevated yolk testosterone levels might also entail costs for these males, which might have manifested as higher resting metabolic rates (Tobler, Nilsson, and Nilsson 2007; Ruuskanen et al. 2013) and subsequently more oxidative damage (Feuerbacher and Prinzinger 1981; Buchanan et al. 2001). Indeed, experimentally increased yolk testosterone has been found to reduce DNA damage repair efficiency (Treidel et al. 2013) and increase telomere shortening (Parolini et al. 2019). Furthermore, experimentally increased yolk testosterone reduced plasma antioxidant levels in male nestlings, but not in female nestlings (Tobler and Sandell 2009). Although we did not measure sex‐specific effects on resting metabolic rate and oxidative damage, we did find several sex‐specific DMS in genes associated with DNA binding, oxidative stress, and DNA repair (Table S20). The sex‐specific effects of elevated yolk testosterone on nestling weight and on DNA methylation of genes related to growth, metabolism, and oxidative damage suggest that there was a trade‐off between investment in nestling growth and investment in DNA repair or protection. Furthermore, these results suggest that the costs and benefits of elevated yolk testosterone differ between sexes, possibly as a result of differences in DNA methylation patterns. It would thus be of particular interest to assess how elevated yolk testosterone affects the covariance between these traits and the covariance between DNA methylation and gene expression of associated genes. We expect that DNA methylation can be an underlying mechanism for mediating sex‐specific trade‐offs across many taxa, as long as the sexes differ in how traits affect their fitness. For example, for males, the costs of elevated yolk testosterone might be outweighed by becoming larger and by subsequently becoming more competitive, thereby increasing their fitness. Whereas for females, there might not be a competitive benefit in being larger.

### 4.4 Future Avenues

Several of the male‐specific and antagonistic DMS were associated with male fertility (Table S19), which is confirmed by experimentally elevated yolk testosterone effects on fecundity and other traits that are important for reproduction, such as plumage development and sexual display (Eising, Muller, and Groothuis 2006; Partecke and Schwabl 2008; Galván and Alonso‐Alvarez 2010), testis and egg size (Uller, Eklöf, and Andersson 2005), and egg fertility and laying activity (Rubolini et al. 2007). This strongly suggests an effect of exposure to high yolk testosterone on fecundity and ultimately fitness via DNA methylation. To confirm this, it would be particularly interesting to assess DNA methylation, sperm quality, and fitness measures, such as lifetime reproductive success, during subsequent breeding seasons.

In addition to assessing effects on cognition, trade‐offs, and reproduction, it would also be interesting to further explore the mechanisms that account for sex‐specific methylation. Sex differences in DNA methylation are known to exist and have been confirmed in humans (Liu et al. 2010; McCarthy et al. 2014; Yousefi et al. 2015; Gatev et al. 2021; Solomon et al. 2022) and chickens (Teranishi et al. 2001; Natt, Agnvall, and Jensen 2014). The mechanisms that account for sex‐specific methylation patterns remain elusive. These differences might be driven by hormones, which could be confirmed with the effects of increased levels of yolk testosterone on DNA methylation in this study. However, we cannot exclude the possibility that genetic variation plays a role too. DNA methylation is not only influenced by environmental factors (Sepers et al. 2021; Sepers, Verhoeven, and van Oers 2024) but also by genetic variation (Höglund et al. 2020; Sepers, Chen, et al. 2023) or by genotype‐by‐environment interactions (Sepers et al. 2019). As the treatment effect on DNA methylation depended on offspring sex, and because the sex chromosomes are highly differentiated in carinate birds (see Solari 1993; Graves 2014), we expect that the epigenetic response to enhanced yolk testosterone will be genotype‐dependent for at least part of the CpGs. In birds, sex is genetically determined, with females being the heterogametic sex (ZW) and males being the homogametic sex (ZZ). Interestingly, none of the DMS was located on the Z chromosome. We potentially missed DMS on the sex chromosomes as we could only analyse a fraction of the CpGs on this chromosome (~0.9%). However, as genetic variation can affect methylation of distant CpGs (Höglund et al. 2020; Sepers, Chen, et al. 2023), it is also possible that genetic variation on the sex chromosomes induces sex‐specific the methylation patterns on the autosomes. To be able to separate maternal effects from sex‐specific genetic effects on DNA methylation, quantitative trait loci should be mapped for the sex‐specific DMS.

## 5 Conclusion

Our study suggests that in contrast to our expectations, experimentally enhanced levels of yolk testosterone resulted in a less competitive phenotype, especially in females. Testosterone affected DNA methylation in genes belonging to different pathways for males and females, pointing to a difference in costs and benefits of being exposed to higher yolk testosterone between the sexes. While it remains challenging to link DNA methylation changes to changes in phenotypic traits, differential methylation of genes involved in metabolism, mass, and development align with the phenotypic effects of elevated yolk testosterone, suggesting that DNA methylation induced by elevated yolk testosterone can affect nestling mass and behaviour. In conclusion, our results support the hypothesis that DNA methylation variation caused by maternal hormones deposited in the egg can be a mediator for sex‐specific effects during early development.

## Author Contributions

K.O., S.R. and B.S. conceived the study. B.S. conducted the experiment and collected all samples and biometry and handling stress data. B.S. and T.M. collected the video recordings and analysed the corresponding data. B.S. conducted the remaining bioinformatic and statistical analyses. A.C.M. conducted the laboratory work. B.S., S.R., and K.O. drafted the manuscript. K.O. supervised the study. All authors contributed to editing the manuscript.

## Ethics Statement

All animal experiments involved in this study were reviewed and approved by the Institutional Animal Care and Use Committee (NIOO‐IvD) and were licensed by the CCD (Central Authority for Scientific Procedures on Animals; AVD‐801002017831) to K.O.

## Conflicts of Interest

The authors declare no conflicts of interest.

## Supporting information


Data S1.

## Data Availability

The raw genomic datasets are deposited on NCBI under BioProject PRJNA208335 under the SRA accessions SRX22027777, SRX22027778, SRX22027779, SRX22027780 and SRX22027781. Data related to brood characteristics and biometry are archived in the SPI‐Birds Database (https://spibirds.org/en; Culina et al. 2021) and can be requested. The epiGBS2 bioinformatics pipeline can be accessed on Github (https://github.com/nioo‐knaw/epiGBS2). All other data sets and scripts are deposited in the Dryad Digital Repository: https://doi.org/10.5061/dryad.k3j9kd5h8.
